# Systematic review of cancer treatment programmes in remote and rural areas

**DOI:** 10.1038/sj.bjc.6690498

**Published:** 1999-06

**Authors:** N C Campbell, L D Ritchie, J Cassidy, J Little

**Affiliations:** Departments of Ge neral Practice and Primary Care and Medicine and Therapeutics, Foresterhill Health Centre, Westburn Road, Aberdeen, AB25 2AY, UK

**Keywords:** cancer treatment, rural areas, patterns of care, systematic review

## Abstract

In an attempt to ensure high quality cancer treatment for all patients in the UK, care is being centralized in specialist centres and units. For patients in outlying areas, however, access problems may adversely affect treatment. In an attempt to assess alternative methods of delivering cancer care, this paper reviews published evidence about programmes that have set out to provide oncology services in remote and rural areas in order to identify evidence of effectiveness and problems. Keyword and textword searches of on-line databases (MEDLINE, EMBASE, HEALTHSTAR and CINAHL) from 1978 to 1997 and manual searches of references were conducted. Fifteen papers reported evaluations of oncology outreach programmes, tele-oncology programmes and rural hospital initiatives. All studies were small and only two were controlled, so evidence was suggestive rather than conclusive. There were some indications that shared outreach care was safe and could make specialist care more accessible to outlying patients. Tele-oncology, by which some consultations are conducted using televideo, may be an acceptable adjunct. Larger and more methodologically robust studies are justified and should be conducted. © 1999 Cancer Research Campaign


*British Journal of Cancer* (1999) **80**(8), 1275–1280
© 1999 Cancer Research Campaign
Article no. bjoc.1999.0498

### Systematic review of cancer treatment programmes in remote and rural areas

**NC Campbell[1], LD Ritchie[1], J Cassidy[2] and J Little[2]**

Departments of [1]General Practice and Primary Care, and [2]Medicine and Therapeutics, Foresterhill Health Centre, Westburn Road, Aberdeen AB25 2AY, UK

**Summary** In an attempt to ensure high quality cancer treatment for all patients in the UK, care is being centralized in specialist centres and units. For patients in outlying areas, however, access problems may adversely affect treatment. In an attempt to assess alternative methods of delivering cancer care, this paper reviews published evidence about programmes that have set out to provide oncology services in remote and rural areas in order to identify evidence of effectiveness and problems. Keyword and textword searches of on-line databases (MEDLINE, EMBASE, HEALTHSTAR and CINAHL) from 1978 to 1997 and manual searches of references were conducted. Fifteen papers reported evaluations of oncology outreach programmes, tele-oncology programmes and rural hospital initiatives. All studies were small and only two were controlled, so evidence was suggestive rather than conclusive. There were some indications that shared outreach care was safe and could make specialist care more accessible to outlying patients. Tele-oncology, by which some consultations are conducted using televideo, may be an acceptable adjunct. Larger and more methodologically robust studies are justified and should be conducted.

**Keywords**: cancer treatment; rural areas; patterns of care; systematic review

The benefits of specialist cancer care are well recognized. Patients cared for by specialists have been reported to receive more up to date treatment, have lower peri-operative mortality rates, fewer recurrences and improved chances of survival, and are more likely to be accrued onto clinical trials (Selby et al, 1996). In recognition of this, the National Health Service in the UK adopted recommendations by the Expert Advisory Group on Cancer in England and Wales (EAGC, 1995) and similar proposals in Scotland (SCCAC, 1996). The structure being developed consists of cancer centres with expertise in all cancers, and cancer units with expertise in common cancers (Haward, 1995). In practice, this means that care is centralized in selected urban locations; in Scotland, all specialist cancer care is provided by five hospitals.

Centralization has unarguable advantages, but also problems. Access, particularly for the fifth of the UK population who live in rural areas (Cox, 1995), is made more difficult. Patients remote from specialist centres have been reported to have later stage diagnoses (Liff et al 1991; Launoy, et al, 1992), less sophisticated treatment (Greenberg et al, 1988*a*; Howe et al, 1992; McCredie et al, 1996; Craft et al, 1997; Kohler et al, 1997) and poorer prognoses (Bonett et al, 1990; Launoy, et al, 1992). Amongst those treated at specialist centres, more distant patients have been found less likely to receive chemotherapy and radiotherapy (Greenberg et al, 1988*b*; Kohler et al, 1997). In future, access problems may not be confined to rural patients. Rapid increases in numbers of patients attending for adjuvant treatments have raised concerns about whether chemotherapy, for example, will be deliverable to all patients who need it by the current structure (Leonard et al, 1997).

*Received 24 August 1998*
*Revised 10 December 1998*
*Accepted 21 January 1999*

*Correspondence to:* NC Campbell

The Expert Advisory Group on Cancer stated that all patients should have access to a uniformly high quality of care wherever they live (EAGC, 1995). For this objective to be met, the means by which cancer care is delivered will need to be examined and, if necessary, reshaped to provide equality of access to modern therapy. The experience of countries with large rural populations have demonstrated the difficulties, but also provided some examples of how this might be attempted (Collins et al, 1997). This paper was written as the first step in a Cancer Research Campaign-funded project on treatment of cancer in rural areas. It sets out to review the literature about programmes providing cancer treatment in remote and rural areas and to identify evidence of effectiveness and problems. Specific questions asked about rural programmes were: can they achieve similar survival rates to specialist centres; can they deliver appropriate treatment to more rural patients; do patients and physicians find them satisfactory; what are their problems (including cost implications)?

### METHODS

Papers were identified from searches of MEDLINE, EMBASE, CINAHL and HEALTHSTAR databases for the period 1978–1997. The search strategy used terms for cancer, such as neoplasms and oncology, and rural, such as rural health and telemedicine, and was supplemented with a textword search (full strategy available from Dr NC Campbell). In addition, relevant citations were followed up.

Papers were eligible if they: (1) described (or cited a paper that described) a programme providing cancer treatment in rural areas; (2) reported a study which aimed to evaluate the programme's effectiveness or identify problems; and (3) came from industrialized countries. All types of evaluation were accepted as long as results (including data) were presented.

### RESULTS

In all, 2697 titles were identified and scanned, and 105 full papers were scrutinized. Fifty-one papers described rural cancer programmes of which 15 described treatment programmes and reported results from evaluations (Tables 1–4). Three papers reported on one programme (Smith DE et al, 1991; Desch et al, 1992; Smith TJ et al, 1996) and two papers on another (Kisker et al, 1980; Strayer et al, 1980), so 15 papers reported on 12 programmes.

Twelve papers (on nine programmes) were from the USA, two were from Australia and one from the UK. They described evaluations with a variety of methods and outcomes: there were no randomized trials, two non-randomized controlled studies (Table 5) and an associated economic evaluation, two before/after uncontrolled studies and ten cross-sectional studies.

Programmes could be divided into four groups: initiatives based at rural hospitals, shared care programmes, outreach programmes and tele-oncology.

### Rural hospital initiatives

Four cross-sectional studies evaluated initiatives based at rural centres (Table 1).

#### *Cross-sectional studies*

In two papers, individual rural general surgeons reported their results. Tulloh and Goldsworthy (1997) audited 3 years of breast surgery. Of 1992 new patients, 275 were seen for breast conditions, of whom 28 had cancer. Twenty-six patients (93%) were managed in consultation with a specialist oncologist. Breast conservation was achieved in 17 (68%) of 25 who had surgery. Chemotherapy was given to 12 patients, initially at a specialist centre, but subsequently by a local specialist nurse under General Practitioner and indirect specialist supervision. Long-term (between 1 and 4 years), four patients who underwent surgery died and one developed metastases. In another paper, Callaghan (1990) audited 20 years of colorectal cancer surgery. Of 168 cases, 119 (71%) were stage C or D. Two patients (1%) died within 30 days of surgery, wound infections occurred in four patients (2%), 5-year survival was 50% overall and 81% for node-free disease. In accompanying commentaries, the results achieved by Tulloh and Callaghan were thought equal to series from specialist centres (Field, 1990; Furnival, 1997). It is difficult to identify features of their practices that could be transferred to other rural areas but their results serve as an encouraging illustration of what can be achieved by some particularly motivated individuals.

Byram et al (1996) reported on the setting up of a provincial radiation oncology service by reviewing treatment statistics from the first year. The main problem identified was higher than anticipated patient turnover (820 patients treated compared with 500 predicted). The authors suggested that improving access had led to more referrals and this should be considered when planning future rural initiatives.

Smith et al (1979) reported on a joint cancer programme between two rural hospitals. The majority of physicians felt the programme was worthwhile. Comparison of cancer surveillance data with other hospitals over time suggested that more patients were being treated locally and there were some indications of better management (e.g. more patients with prostate cancer were receiving radiotherapy).

### Shared care with central clinics

Two papers reported on a shared care programme for children with cancer (Table 2). Specialists at a university centre were responsible for diagnosis and assigning treatment protocols, but 70% of care, including monitoring and chemotherapy administration, was conducted by nearby family or paediatric practitioners.

**Table 1** Rural hospital initiatives

| Author | Place | Aims of paper as stated in introduction or abstract | Type of programme | Type of evaluation | Evaluation outcomes | Numbers and response rates |
|---|---|---|---|---|---|---|
| Tulloh and Goldsworthy, 1997 | Victoria, Australia | To describe how breast cancer is managed in the practice of a general surgeon in a rural town. | Rural surgical practice. | Cross-sectional review of medical records. | Patterns of treatment (surgery, radiotherapy, chemotherapy), involvement of oncologists, complications and long-term outcome. | All 28 patients with breast cancer in a 3-year period. |
| Callaghan, 1990 | Iowa, USA | To report a surgeon's experience with colorectal cancer over a 20-year period in a small rural hospital. | Rural surgical practice | Cross-sectional review of treatment records. | Stage at diagnosis, 5-year survival, postoperative deaths and complications. | 168 cases. |
| Byram et al, 1996 | Victoria, Australia | To report the workload experience in the first 12 months. | Provincial radiation oncology service. | Cross-sectional review of treatment records. | Patterns of radiation treatment, diagnoses of patients, concurrent chemotherapy, population demographics, numbers entered on trials. | 1009 patients |
| Smith et al, 1979 | Washington State, USA | To document the impact of a hospital cancer programme on the delivery of care to cancer patients. | Hospital cancer programme in a rural county . | Cross-sectional study. | Patterns of care, physician and consultant satisfaction. | Two programme and five other hospitals (4843 cancer registrations), 90 physicians surveyed, 65 (72%) responded; 22 consultants surveyed, all responded. |

*© 1999 Cancer Research Campaign*

**Table 2** Shared care with central clinics

| Author | Place | Aims of paper as stated in introduction or abstract | Type of programme | Type of evaluation | Evolution outcomes | Numbers and response rates |
|---|---|---|---|---|---|---|
| Kisker et al, 1980 | Iowa, USA | To evaluate selected medical outcomes provided by the shared management system. | Community-based care programme for children with cancer. | Non-randomized controlled study. | Patient outcomes (febrile episodes, infections, drug toxicities, neutropenia, thrombocytopenia, hospitalization) and physician performance (protocol non-compliance, non-reporting the six patient outcome factors) | 46 eligible patients out of 82 with cancer. Data presented on all 46. |
| Strayer et al, 1980 | Iowa, USA | To evaluate the potential cost differences between the shared-management system and the specialist approach. | Community-based shared-care programme for children with cancer. | Economic evaluation. | Direct and indirect costs. | 16 patients attending the shared management system. |

**Table 3** Shared care with outreach clinics

| Author | Place | Aims of paper stated in introduction or abstract | Type of programme | Type of evaluation | Evaluation outcomes | Numbers and response rates |
|---|---|---|---|---|---|---|
| Howe et al, 1997 | Illinois, USA | To compare an intensive rural oncology outreach programme with a lower intensity physician education programme. | Intensive oncology outreach and lower intensity physician education programmes. | Non-randomized controlled study. | Breast cancer management practices. | 817 cases with breast cancer in 1990–1. Case notes of >99% were followed up. |
| Smith et al, 1996 | Virginia, USA | To evaluate outcomes and perform a financial analysis. | Rural oncology outreach programme. | Before/after uncontrolled study treatment. | Patterns of breast cancer treatment, clinical trial accrual and use of morphine. | Not reported |
| Hammond et al, 1987 | Montana, USA | (To report effects on clinical trial accruals.) | Community clinical oncology programme. | Before/after uncontrolled study | Patient characteristics, changes in data management, changes in accruals. | 432 patients pre CCOP and 222 patients post CCOP. |
| White et al, 1996 | Michigan, USA | To examine the impact of the advanced practice nurse on cancer patient education in an outpatient setting. | Advanced practice cancer nurses. | Cross-sectional review of clinic data. | Initial patient knowledge deficit, diagnoses and nursing interventions. | 170 cases. All reviewed. |
| Grose et al, 1995 | Stockport, UK | To investigate the impact of a urological community nurse on practice, efficiency and quality of care. | Urological community nurse. | Cross-sectional review of procedures undertaken in 1 year. | Procedures. | One community nurse. 464 procedures. |
| Guy et al, 1988 | Ohio, USA | (To assess financial viability of in-patient admissions from rural outreach clinics.) | Rural oncology outreach programme. | Cross-sectional review of clinic data. | Diagnostic and admission characteristics, charges and reimbursements. | 94 patients attending two outreach clinics. |

### *Non-randomized controlled study*

Kisker et al (1980) compared health outcomes of 24 children receiving shared care with 22 children who received specialist care at another university centre. Both centres used the same treatment protocols. Seventeen eligible patients declined to participate from preference or convenience (12 eligible for intervention and five control). No significant differences between groups were reported in febrile episodes and infections, drug toxicity, blood dyscrasias or protocol compliance. Slight differences in recording (e.g. platelet counts) were not thought to be clinically significant. The study had considerable methodological limitations (Table 5), most importantly that numbers of patients were small so only large differences could have been detected. The study has, then, demonstrated the feasibility of shared care, but larger studies are needed to show its safety

Strayer et al (1980) analysed costs for 16 intervention-group patients in the same study and compared them with postulated costs had they been treated at the specialist centre. Direct medical costs were similar, but there were savings of approximately $2000 (US) per patient in other direct costs (mostly reduced transport costs) and indirect costs (lost productivity). This represented 41% of total standard care costs.

### Shared care with outreach clinics

There were six papers about outreach programmes, in which specialists from urban centres travelled to rural centres at regular intervals (Table 3). The frequency of clinics was not always specified, but could be as often as weekly or fortnightly (Guy et al, 1988; Desch et al, 1992). Care between visits was by local practitioners, often supported by specialist nurses.

*© 1999 Cancer Research Campaign*

**Table 4** Shared care with tele-oncology clinics

| Author | Place | Aims of paper as stated in introduction or abstract | Type of programme | Type of evaluation | Evaluation outcomes | Numbers and response rates |
|---|---|---|---|---|---|---|
| Allen and Hayes, 1955 | N Carolina, USA | To evaluate patient satisfaction with tele-oncology consultations. | Telemedicine oncology outreach | Cross-sectional study. | Patient satisfaction. | 39 patients completed questionnaire. 21 (54%) were followed up on site. |
| Allen et al, 1955 | N Carolina, USA | A pilot study of the physician satisfaction with tele-oncology clinics. | Telemedicine oncology outreach. | Cross-sectional study. | Physician satisfaction. | Three oncologists completed forms after 34 consultations. On-site follow-up forms were completed for seven patients. |
| Doolittle et al, 1997 | N Carolina, USA | To examine the cost of providing tele-oncology, outreach cancer and hospital-based traditional oncology services. | Telemedicine oncology outreach. | Cost analysis based on cross-sectional study. | Health service costs. | 103 tele-oncology and 81 outreach visits. Numbers of hospital-based traditional visits not stated. |

**Table 5** Methodological features of non-randomized controlled studies

| Study | Basis of group allocation | Baseline differences in comparison groups | Adjustments in analysis | Study power |
|---|---|---|---|---|
| Kisker et al, 1980 | Intervention group: patients attending University of Iowa. Control group: patients attending University of Cincinnati. | 14 of 24 (58%) intervention and eight of 22 (41%) control patients had leukaemia (the remainder had solid tumours). Patient characteristics and severity of disease at diagnosis were not described. | Results from patients with leukaemia and solid tumours were analysed separately. | Not reported, but likely to be low (comparison groups had only eight and 14 patients each). |
| Howe et al, 1997 | Rural group 1: patients attending five rural hospitals in Illinois. Rural group 2: patients attending four rural hospitals in Illinois. Comparison group: urban patients attending four urban hospitals in Illinois. | Breast cancer management practices of both rural groups at baseline were similar (58% of both received state-of-the-art care). Patient characteristics and disease stage at diagnosis were not described | Logistic regression was used to adjust for stage at diagnosis and baseline levels of each management practice. | Not reported. Rural groups 1 and 2 had 67 and 105 patients respectively. The urban comparison group had 499 patients. |

### Non-randomized controlled study

Howe et al (1997) reported two approaches to rural breast cancer care. Five hospitals received an intensive oncology outreach programme coupled with education for local clinicians based on audit feedback and four other hospitals received only the education component. Urban patients attending urban hospitals were used as a comparison group. At baseline, state-of-the-art care (according to National Cancer Institute guidelines) was achieved for 58% of patients in both rural groups compared to 70% in the urban group. At outcome, it was achieved for 63% of 105 patients at hospitals with outreach and 55% of 67 patients at hospitals with education. Only the latter remained significantly worse than the urban group (71% of 449 patients, $P < 0.01$).

### Before/after uncontrolled studies

Smith et al (1996) reported a chart audit 2 years before and 3 years into a cancer outreach programme (Smith TJ et al, 1991; Desch et al, 1992). At one rural site, the proportion of chemotherapy delivered locally increased from 0% to nearly 100% and significantly more breast cancer patients had tumour size recorded (59% vs 29%, $P = 0.03$) and breast conservation (70% vs 20%, $P = 0.004$). Overall, the number of patients from the served rural areas under specialist/outreach care increased by 330%. Assessing the overall effect of this programme is, however, difficult. Patient care was reported for only one of three rural centres, and local care was studied despite most patients receiving at least some central care. Patterns of care would have been expected to change in a similar direction around this time, so how much was due to the outreach programme is not clear.

Hammond et al (1987) reported the effects of a community clinical oncology programme on clinical trial accruals. Clinics were established in communities of more than 10 000 people. They were evaluated by analysing hospital admission registers and databases of patients entered on national studies before and after the programme started. Overall, patient accrual increased by 25% with a higher proportion from outlying areas.

### Cross-sectional studies

Two studies set out to examine the impact of specialist nurses in rural communities. White et al (1996) reviewed clinic data on 170 patients who attended ambulatory nurse-operated satellite clinics run as an adjunct to specialist cancer care; they identified common knowledge deficits and symptoms. Grose et al (1995) reviewed 464 procedures undertaken by a urological community nurse in 1 year. The nurse conducted 33 mitomycin instillations for bladder cancers and assisted in the management of one patient with terminal prostate cancer whose catheter was prone to blockage. Despite their aims, however, neither study assessed the effectiveness of their nurse programmes so little can be concluded. Guy et al (1988) reviewed clinic data of 94 patients attending two oncology outreach clinics (of whom 77 had cancer) to assess charges and reimbursement and found that their outreach clinics served less affluent populations with less capacity to pay.

 © 1999 Cancer Research Campaign

### Shared care with tele-oncology clinics

Three papers reported on a tele-oncology programme (Table 4). This variation on outreach has patients at remote locations consulting with specialists by televideo. Day to day care is shared with local practitioners.

#### *Cross-sectional studies*

In two papers, Allen et al (1995*a*, 1995*b*) reported on patient and physician satisfaction with tele-oncology consultations. At the remote site, patients were accompanied by an oncology nurse practitioner, who presented the case and acted as surrogate examiner. Overall patient satisfaction with tele-oncology consultations was reasonably high, although it declined slightly after in-person follow-up. Physician satisfaction was also reasonably high. Numbers in both studies were small.

Doolittle et al (1997*a*) monitored costs for three types of oncology practice: a telemedicine clinic; a fly-in outreach clinic; and a traditional city clinic for 1 year. Only direct health service costs were included in the analysis. The average cost per telemedicine visit was $812, outreach oncology visits were $897 and traditional clinic visits were $149. The estimated costs for telemedicine visits included start-up costs; the projected cost if the system was at full capacity was $301. Neither direct nor indirect patient costs were included in the analysis.

### DISCUSSION

### Shortcomings

In this review, the total number of rural cancer care programmes identified was small and less than a third had been evaluated. This seems to confirm the known paucity of research in rural areas (Cox, 1995). It is also possible that some papers on rural cancer care were not identified by our search: the search strategy employed was broad, but for programmes to be eligible, they had to state that they were rural or remote and served a rural population; some rural programmes may not have done so. Similarly, community oncology programmes were eligible only if they stated that they served a rural population. The USA has a large network of community clinical oncology programmes but they tend to be concentrated in areas of high population density (Kaluzny et al, 1989; Cobau, 1994), so few were eligible.

All studies had methodological limitations. Only two had control groups (Kisker et al, 1980; Howe et al, 1997) and, in them, numbers were small, designs open to bias and adjustment for confounding factors incomplete (Table 5). Their statistical power, particularly to demonstrate that a programme was not worse than specialist care, was limited. The outcome measures used varied widely between studies but were mostly intermediate (patient satisfaction, physician performance etc.). Only three papers reported effects on patient health or survival (Kisker et al, 1980; Callaghan, 1990; Tulloh and Goldsworthy, 1997). Overall, therefore, the evidence in this review is at best suggestive, and should be viewed as a platform for more methodologically robust research, rather than the basis for changes in clinical practice.

### Relevance to the UK

There was little evidence from the UK, so relevance is limited and indirect. Comparing the findings of different studies and relating them to other rural areas is difficult because rural settings vary. There are few similarities, for example, between remote towns in rural Australia and villages in England. Most programmes in this review were set in the USA and cared for patients in rural towns that were remote from specialist services so they are, perhaps, most relevant to these areas. Even there, it is possible that any effect might be confined to patients who lived near the local ‘centre’ and less relevant in other areas. In rural ‘centres’, local practitioners were often general physicians or surgeons. There was less evidence about care for patients remote from rural towns, whose only local doctor is likely to be their general practitioner.

### CONCLUSIONS

Programmes that have attempted to provide high quality cancer treatment in rural areas vary from rurally driven to centrally based initiatives. Some of the former appear to have demonstrated that high quality cancer care is possible, at least in rural centres. Numbers in these series were, however, relatively small and most rural centres do not achieve the outcomes reported by Tulloh and Callaghan. When breast cancer management in the USA and Australia was assessed by indicators such as breast conservation, rural hospitals performed poorly (Howe et al, 1995; Craft et al, 1997). Similarly, prostate cancer treatment was reported to be 5 years out of date (McCredie et al, 1996). In the absence of particularly interested local practitioners it seems unlikely that improvements can be achieved without specialist involvement.

One paper reported on a rural radiotherapy centre (Byram et al, 1996). They suggested (although did not prove) that better access exposed hidden demand. The setting was rural Australia, however, where distances are vast and the catchment of 500 000 was not particularly small. In the UK, Penn (1992) has reported on a radiotherapy facility in Torbay (catchment 250 000). It achieved similar outcomes to those of main centres, with better patient convenience. Numbers of cases were, however, small and problems (e.g. capital outlay and staff recruitment) were identified. These papers are about the size of town that justifies radiotherapy. Rural patients have no option but to travel.

There is some evidence that a shared approach between specialists and local practitioners may be the way forward. It has proved possible for rural practitioners to take on a proportion of routine monitoring and chemotherapy administration. There is some evidence that this is an improvement on local non-specialist care, but it has not yet been shown convincingly to be better than travelling to specialist centres. Nor is it clear how specialists should consult in a shared care system, although we have some idea of the cost implications (Doolittle et al, 1997). Outreach clinics were the least economically attractive, with a sixfold increase in cost per visit in one study, so could only be justified if there were considerable and demonstrable patient benefits. Tele-oncology clinics were cheaper than outreach, but at least double the cost of central clinics. More evidence is needed about their acceptability and effects on patient outcomes. Limited experience in Scotland has been encouraging (Kunkler et al, 1997), but anecdotal reports suggest limitations: some patients were less satisfied, particularly with first consultations; some physicians found the system more difficult than others and there were concerns about breaking bad news (Doolittle and Allan, 1997). Clearly, this requires further study.

It is not possible from this review to make recommendations for the provision of cancer services in remote and rural areas. The

© 1999 Cancer Research Campaign review does, however, point out the priorities for further research. First, existing studies of shared care are not conclusive and effects on patients' health, quality of life and survival require further description. Secondly, it is not known whether rural practitioners are motivated to take on the responsibility of shared care oncology, nor how safe it would be in the hands of less enthusiastic practitioners. Finally, the benefits and disadvantages of tele-oncology over central clinics need to be evaluated. In the future, models of care should ideally be tested using more robust methods, preferably randomized trials.

### ACKNOWLEDGEMENTS

We thank the Cancer Research Campaign who fund Neil Campbell's Fellowship. Thanks also to Cynthia Fraser of the Cochrane Centre who helped develop the search strategy.

### REFERENCES

Allen A and Hayes J (1995) Patient satisfaction with teleoncology: a pilot study. *Telemed J* **1**: 41–46

Allen A, Hayes J, Sadasivan R, Williamson SK and Wittman C (1995) A pilot study of the physician acceptance of tele-oncology. *J Telemed Telecare* **1**: 34–37

Bonett A, Dorsch M, Roder D and Esterman A (1990) Infiltrating ductal carcinoma of the breast in South Australia. Implications of trends in tumour diameter, nodal status and case-survival rates for cancer control. *Med J Aust* **152**: 19–23

Byram J, Joseph D and Bulmer M (1996) The development of a provincial radiation oncology service: the first year report and its implications for future developments. *Austl Radiol* **40**: 306–309

Callaghan J (1990) Colorectal cancer in a small rural hospital. *Am J Surg* **159**: 277–280

Cobau CD (1994) Clinical trials in the community. The community clinical oncology program experience. *Cancer* **74**: 2694–2700

Collins JP (1997) 'Best practice' in surgical management of breast cancer. *Med J Aust* **166**: 620–621

Cox J (1995) *Rural General Practice in the United Kingdom. Occasional Paper 71*. The Royal College of General Practitioners: London

Craft PS, Primrose JG, Lindner JA and McManus PR (1997) Surgical management of breast cancer in Australian women in 1993: analysis of Medicare statistics. *Med J Aust* **166**: 626–629

Desch CE, Smith TJ, Breindel CL, Simonson CJ and Kane N (1992) Cancer treatment in rural areas. *Hosp Health Services Admin* **37**: 449–463

Doolittle GC and Allen A (1997) Practising oncology via telemedicine. *J Telemed Telecare* **3**: 63–70

Doolittle GC, Harmon A, Williams A, Allen A, Boysen CD, Wittman C, Mair F and Carlson E (1997) A cost analysis of a tele-oncology practice. *J Telemed Telecare* **3**: 20–22

Expert Advisory Group on Cancer (1995) *A Policy Framework for Commissioning Cancer Services*: *a Report by the Expert Advisory Group on Cancer to the Chief Medical Officers of England and Wales, 1995*. Department of Health. HM Stationery Office: London

Field RJ Jr (1990) Editorial comment. *Am J Surg* **159**: 281

Furnival CM (1997) Breast cancer in rural Australia. *Med J Aust* **166**: 25–26

Greenberg ER, Dain B, Freeman D, Yates J and Korson R (1988*a*) Referral of lung cancer patients to university hospital cancer centers. *Cancer* **62**: 1647–1652

Greenberg ER, Chute CG, Stukel T, Baron JA, Freeman DH, Yates J and Korson R (1988*b*) Social and economic factors in the choice of lung cancer treatment. A population-based study in two rural states. *N Engl J Med* **318**: 612–617

Grose K, Brooman PJC and O'Reilly PH (1995) Urological community nursing: a new concept in the delivery of urological care. *Br J Urol* **76**: 440–442

Guy J, Imhoff M and Coffman CA (1988) Financial viability of in-patient admissions from rural oncology outreach clinics. *Prog Clin Biolog Res* **278**: 219–225

Hammond N, Marchello B, Myers D and Kampen S (1987) Evaluation of the impact of a CCOP program in a low population density area of Montana. *Prog Clin Biol Res* **248**: 205–212

Haward RA (1995) Establishing cancer units. *Br J Cancer* **72**: 531–534

Howe HL, Katterhagen JG, Yates J and Lehnerr M (1992) Urban–rural differences in the management of breast cancer. *Cancer Causes Control* **3**: 533–539

Howe HL, Johnson TP, Lehnerr M, Warnecke RB, Katterhagen G and Ford L (1995) Patterns of breast cancer treatment: a comparison of a rural population with an urban population and a community clinical oncology program sample. *Cancer Control* **2**: 113–120

Howe HL, Lehnerr M and Katterhagen G (1997) Effects of physician outreach programs on rural–urban differences in breast cancer management. *J Rural Health* **13**: 109–117

Kaluzny AD, Ricketts T 3d, Warnecke R, Ford L, Morrisey J, Gillings D, Sondik EJ, Ozer H and Goldman J (1989) Evaluating organizational design to assure technology transfer: the case of the Community Clinical Oncology Program. *J Natl Cancer Inst* **81**: 1717–1725

Kisker CT, Strayer F, Wong K, Clarke W, Strauss R, Tannous R, Janco R and Spevak J (1980) Health outcomes of a community-based therapy program for children with cancer – a shared management approach. *Pediatrics* **66**: 900–906

Kohler CRD, Keys SD, Hutcheon AW, Walker LG and Eremin O (1997) Do sociodemographic factors influence the decision to give chemotherapy to women with breast cancer? *Br J Surg* **84**: 46

Kunkler IH, Rafferty P, Hill DM, Henry M and Foreman D (1997) Telemedicine. Proved acceptable in pilot study in oncology in Scotland. *Br Med J* **314**: 521

Launoy G, Le Coutour X, Gignoux M, Pottier D and Dugleux G (1992) Influence of rural environment on diagnosis, treatment and prognosis of colorectal cancer. *J Epidemiol Community Health* **46**: 365–367

Leonard RCF, Smith IE, Coleman RE, Malpas JS, Nicolson M, Cassidy J, Jones A, McIllmurray MB, Stuart NSA, Woll PJ and Whitehouse JMA (1997) More money is needed to care for patients with cancer. *Br Med J* **315**: 811–812

Liff JM, Chow WH and Greenberg RS (1991) Rural–urban differences in stage at diagnosis. Possible relationship to cancer screening. *Cancer* **67**: 1454–1459

McCredie M, Bell J, Lee A and Rogers J (1996) Differences in patterns of care of prostate cancer. *Aust NZ J Surg* **66**: 727–730

Penn CRH (1992) Megavoltage irradiation in a district general hospital remote from a main oncology centre: the Torbay experience reviewed. *Clin Oncol* **4**: 108–113

Scottish Cancer Co-ordinating and Advisory Committee (1996) *Commissioning Cancer Services in Scotland*: *Report to the Chief Medical Officer, Scottish Office Department of Health*. HM Stationery Office: Edinburgh

Selby P, Gillis C and Haward R (1996) Benefits from specialised cancer care. *Lancet* **348**: 313–318

Smith DE, Davis S and Polissar L (1979) The hospital cancer program: its impact on care of the rural cancer patient. *Am Surg* **45**: 730–737

Smith TJ, Desch CE, Simonson CJ and Kane N (1991) Teaching specialty cancer medicine in rural hospitals: the cancer outreach program as a model. *J Cancer Educ* **6**: 235–240

Smith TJ, Desch CE, Grasso MA, McCue MJ, Buonaiuto D, Grasso K, Johantgen ME, Hackney MH, Shaw JE and Simonson CJ (1996) The rural cancer outreach program: clinical and financial analysis of palliative and curative care for an underserved population. *Cancer Treatment Rev* **22**: 97–101

Strayer F, Kisker T and Fethke C (1980) Cost-effectiveness of a shared-management delivery system for the care of children with cancer. *Pediatrics* **66**: 907–911

Tulloh BR and Goldsworthy ME (1997) Breast cancer management: a rural perspective. *Med J Aust* **166**: 26–29

White N, Given BA and Devoss DN (1996) The advanced practice nurse: meeting the information needs of the rural cancer patient. *J Cancer Educ* **11**: 203–204

 *© 1999 Cancer Research Campaign*
